# Correlation between TLR9 Expression and Cytokine Secretion in the Clinical Diagnosis of Systemic Lupus Erythematosus

**DOI:** 10.1155/2015/710720

**Published:** 2015-09-17

**Authors:** Hui Rao, Qinghua Zeng, Yumei Liang, Changjuan Xiao, Shuoshan Xie, Xiangyu Xu

**Affiliations:** The People's Hospital of Hunan Province, Rheumatism Immunity Branch, Changsha 410002, China

## Abstract

To investigate the correlation between TLR9 and cytokine secretion in SLE diagnosis and treatment. A total of 66 cases (39 SLE and 27 healthy donors) were enrolled in this study. The CD20+ labeled B cells were isolated from SLE patients. TLR9 mRNA expression from SLE tissues and B cells was detected using RT-PCR. The cytokine secretion in B cells were measured using ELISA. Correlation between TLR9 expression and cytokines secretion was analyzed using gene silencing method. Compared with the controls, TLR9 expression was significantly high in SLE patients tissues, as well as in B cells. Expressions of IL-6 and ds-DNA antibody were high in SLE patients serum and were positively correlated with TLR9 level in SLE patients (IL-6, *R* ^2^ = 0.768; ds-DNA antibody, *R* ^2^ = 0.730). The IL-6 and ds-DNA expression were significantly decreased by silencing TLR9 compared to the controls. Moreover, silencing TLR9 significantly decreased cytokines secretion including IL-6, IL-10, and IL-1r*α*, as well as the pathway-associated protein expression, including ICOS and Foxp3. The successful application of TLR9 silencing method in human SLE B cells may loan theatrical basis for the possibility of TLR9 genetic therapy in SLE diagnosis and treatment.

## 1. Introduction

Systemic lupus erythematosus (SLE) is a common autoimmune disease, which is characterized by the abnormal activation of self-reactive T and B cells and production of self-antibodies and immune complex, and can lead to irreversible damage to organ systems [1]. Complications such as osteoporosis, urinary system diseases, and pseudo obstruction that resulted from SLE have brought huge damage for patients' life [2, 3]. Even though several diagnostic criteria have been made for SLE diagnosis in clinical and played certain significant roles in SLE treatment and diagnosis, the results for SLE diagnosis still remain unsatisfactory due to the diversity and hidden characteristics, serology indexes, and complicate pathogen mechanism [4, 5]. Therefore, it will be of great significance to investigate the mechanism for SLE treatment.

Previous viewpoint shows that B-cell activation in SLE patients is mainly assisted by T cells [6], but recent reports indicate that B-cell activation in SLE development depends on the activation of Toll-like receptors (TLRs) pathway instead of T cell [7]. TLRs are some signal transduction receptors for pattern recognition receptors which can recognize various kinds of ligands and specific molecular patterns and can induce some fight infection and innate immunity and then play pivotal roles during innate immunity [8, 9]. TLR9 is a member of the TLR family which plays a fundamental role in pathogen recognition and activation of innate immunity [10]. Recent papers show that several TLR members play certain roles in SLE progression and pathogen, including TLR9 and TLR7 [11]. Ligands for TLR9 are some nonmethylate or low methylate DpG DNAs and play certain recognition roles in endoplasmic reticulum, which is different from other receptors [12]. Meanwhile, increasing evidence shows that many TLRs-induced cytokines play crucial toxic actions in the development of SLE, and the combination of TLR9 and ligand could activate variety of immune cells to produce cytokines. For instance, when pDC from female B6 129F2 rats was stimulated by HSV-2 DNA, IFN-*α* secreted by TLE9−/− cell was significantly declined compared with that in wild type rats [13]. Also, mRNA expression of TLR9 in active stage SLE is high, as well as the IL-10 level in SLE PBMCs, which suggests the correlation between TLR9 level and IL-10 expression in SLE [14].

Although many studies have been devoted to the mechanism exploration of TLRs in SLE development or diagnosis, roles of TLR9 in clinical SLE diagnosis still remain controversial. In this study, we used comprehensive experimental methods to detect the expression of TLR9 and to analyze the correlation between TLR9 expression and cytokines secretion level in SLE patients. This study aimed to illustrate the correlation between TLR9 expression and SLE pathogen mechanism. Our study may provide basis for TLR9 application in the clinical diagnosis and treatment for SLE.

## 2. Materials and Methods

### 2.1. Patients and Samples

A total of 39 patients who were diagnosed as SLE in the Third Xiangya Hospital Central South University and with at least 2 years of follow-up were enrolled in this study. Thereto, 9 are male and 30 are female, 26 patients were complicated with nephritis, 9 cases were complicated with pulmonary lesions, 12 cases were complicated with heart damage, and 15 patients were complicated with skin lesion. Also, 27 cases were in active stage while 12 were in remittent stage. There was no significant difference of age among selected patients. The characteristics of the total enrolled SLE patients and healthy controls are summarized in Table 1.

Patients who suffered from SLE were diagnosed based on the American College of Rheumatology (ACR) 1997 revised criteria [15]. Healthy persons were included according to the normal physical examination standard in this hospital. All the experimental procedures in this study were approved by the ethics committee from the Third Xiangya Hospital Central South University, and informed consent and approval of patients were obtained from all the enrolled patients.

The SLE tissue samples were collected at the time of surgery, snap-frozen in liquid nitrogen, and then stored at −80°C. For peripheral blood B-cell isolation, 5 mL of peripheral blood was labeled with 50 *μ*L of anti-human CD20 antibody supplemented with colloidal paramagnetic microbeads (Miltenyi Bio, Germany) [16]. Isolated B cells with purity of >95% were assessed by flow cytometry [17].

### 2.2. Real-Time- (RT-) PCR Analysis

TLR9 mRNA expression in B cells from SLE patients or healthy persons was detected as previously described [18]. Briefly, B cells isolated from different human skin tissues were grinded in cell lysis buffer and then washed with PBS buffer for 3 times. Total RNA from B cells was extracted using Trizol extraction reagent (Invitrogen, USA) according to manufacturer's protocol [19], and then RNase-free Dnase I (Sangon, Shanghai, China) was added to mixtures to remove DNA. The concentration and purity of isolated RNA were measured using SMA4000 UV-VIS (Merinton, Shanghai, China) at the wavelength of 260 nm. The purified RNA (0.5 *μ*g/*μ*L) was used for cDNA synthesis with the PrimerScript 1st Strand cDNA Synthesis Kit (Invitrogen, USA). Expressions of mRNAs were detected using SYBR green-based quantitative RT-PCR (Invitrogen, USA). The total reaction system (20 *μ*L volume) containing 1 *μ*L cDNA from the above PCR, 10 *μ*L SYBR Premix EX Taq, 1 *μ*L each of the primers (10 *μ*M), and 7 *μ*L ddH_2_O. PCR reaction was carried out at 50°C for 2 min and 95°C for 10 min followed by 40 cycles of 95°C for 15 s and 60°C for 1 min. Melting curve analysis of amplification products was performed at the end of each PCR to confirm that only one product was amplified and detected. In addition, *β*-actin was chosen as the internal control. Primers used for targets amplification were shown as follows: TLR9 sense primer: 5′-ATGGGTTTCTGCCGC-3′, antisense primer: 5′-GGACGCCGTAGAGAAG-3′; Foxp3 sense primer: “CTCAAGCACTGCCAGGCGGAC”, and antisense primer: “CAGCGGATGAGCGTGGCGTAGG”; *β*-actin sense primer: 5′-GGACTTCGAGCAAGAGATG-3′, antisense primer: 5′-ACATGCGGTTGTGTCACGA-3′. In addition, TLR9 antibody (Invitrogen, USA) was labeled with fluorescein isothiocyanate (FITC), and CD20 antibody labeled B cells were used to determine the expression of TLR9 in B cells as previously described [14].

### 2.3. Enzyme-Linked Immune Sorbent Assay (ELISA)

Ten mL of peripheral blood isolated from SLE patients and healthy donors was centrifuged at 12,000 rpm at 4°C to obtain serum. Serum anti-ds-DNA antibody and serum IL-6 were detected using ELISA kit (RD, USA) as previously described [20, 21]. Briefly, pretreated microtitre plates were coated with 50 *μ*g/mL of calf thymus dsDNA (Sigma, USA) for 2 h at 37°C and then placed overnight at 4°C. After that, plates were washed with PBS buffer containing 0.05% Tween-20 (PBST) for three times and then blocked with 5% goat serum in PBST for 1 h. Then serum samples (diluted at 1 : 100 in PBST containing 10% calf serum and 5% goat serum) were incubated with horseradish peroxidase- (HRP-) conjugated goat anti-mouse IgG (Sigma, USA) for 2 h at 37°C. Finally, reaction was blocked with 2 N H_2_SO_4_ and absorbance was measured using a microplate reader (BioRad) at 492 nm.

### 2.4. Correlation Analysis between TLR9 Expression and Cytokines

siRNA-TLR9 (Invitrogen, USA) plasma was transfected into CD20+ labeled B cells for 24 h. B cells transfected with scrambled siRNA plasma were considered as the controls. Total RNA was isolated from the B cells in each group based on the Trizol extraction reagent (Invitrogen, USA) according to manufacturer's protocol [19]. Consequently, relative TLR9 mRNA expression in B cells transfected with siRNA-TLR9 plasma was measured based on the RT-PCR analysis which was described in the above method. Primers used for silencing TLR9 were sense primer: 5′-GAAAGCATCAACCACACCAA-3′, antisense primer: 5′-ACAAGTCCACAAAG CGAAGG-3′. PCR was conducted using the protocol of 30 cycles of 94°C for 30 s, 56°C for 30 s, and 72°C for 30 s. D-Glyceraldehyde-3-phosphate dehydrogenase (GADPH) was used as internal control.

Additionally, expressions of IL-6 and ds-DNA antibody in B cells transfected with siRNA-tlr9 were detected using ELISA as described in Section 3 (part three of Methods).

### 2.5. B-Cell Stimulation In Vitro

B cells were stimulated in vitro with CpG 2006 oligonucleotide (CpG) (TIB MolBiol Synthese Labor GmbH, Berlin, Germany). The cells were resuspended in RPMI 1640 Glutamax supplemented with 10% FBS, 5% penicillin/streptomycin, and 0.05 mM 2-mercaptoethanol (Gibco Life Technologies GmbH, Darmstadt, Germany). B cells at 1 × 10^5^ were seeded and stimulated with 2.5 *μ*g/mL CpG for 48 h at 37°C. After 2 days of culture, the supernatants were harvested and frozen at −70°C prior to analysis.

### 2.6. Statistical Analysis

All experiments were conducted three times independently in this study. The total presented data were firstly tested for the normal distribution using one-sample *K*-*S* test and were expressed as mean ± standard error of mean (SEM). Difference of the data was analyzed using the graph prism 5.0 software (GraphPad Prism, San Diego, CA). Independent sample *t*-test was used to calculate the difference between two groups. ANOVA (analysis of variance) was used to calculate the difference for more than 3 groups. *P* < 0.05 was considered as statistically significant.

## 3. Results

### 3.1. Expression of TLR9 in B Cell from SLE Patients

**Figure 1:**
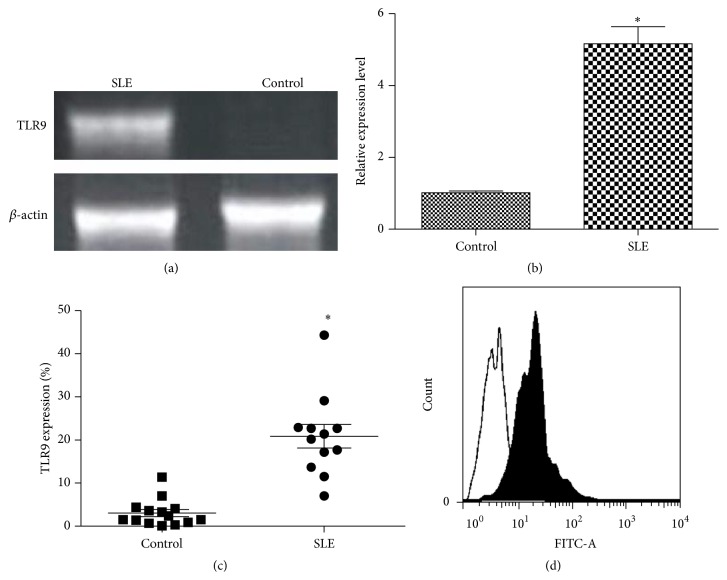
Expression of TLR9 in SLE patients. (a) RT-PCR analysis of TLR9 expression in SLE tissue from patients; (b) mRNA expression of TLR9 in tissues from SLE patients; (c)-(d) flow cytometry analysis of TLR9 expression in B cell; expression of TLR9 in control cells was 4.0% but in SLE cells was 21.2%. ^*∗*^ *P* < 0.05, compared with the controls.

RT-PCR analysis was used to detect the expression level of TLR9 in B cells from SLE patients (Figure 1). TLR9 mRNA level in SLE patients was significantly increased compared with that in healthy patients (*P* < 0.05, Figures 1(a) and 1(b)). Additionally, flow cytometry analysis indicated that TLR9 level in CD20+ B cells from SLE patients was significantly higher than that in control B cells (*P* < 0.05, Figures 1(c) and 1(d)).

### 3.2. IL-6 and ds-DNA Antibody Assay in SLE Patients

**Figure 2:**
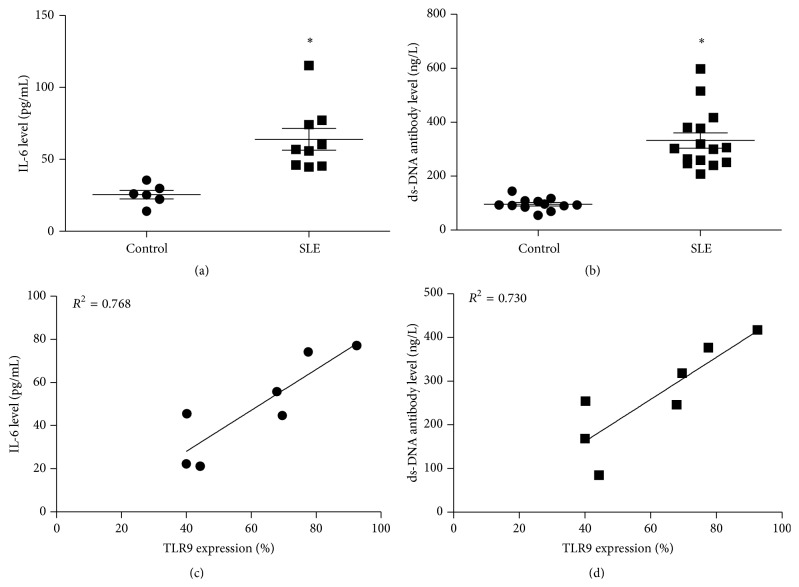
Correlation analyses between TLR9 expression and cytokines level in SLE patients. (a)-(b) IL-6 and ds-DNA antibody level in SLE patients; (c)-(d) correlation between TLR9 overexpression and IL-6 or ds-DNA antibody level. ^*∗*^ *P* < 0.05, compared with the controls.

Th2 cytokines such as IL-6 and IL-10 have been demonstrated to be involved in SLE development [22, 23]. Flow cytometry was used to assess the correlation between TLR9 expression and cytokines secretion in B cells isolated from SLE patients (Figure 2). Compared with the controls, IL-6 expression and ds-DNA antibody level in SLE patients were significantly increased (*P* < 0.05, Figures 2(a) and 2(b)). However, when we analyzed the correlations between TLR9 expression and IL-6 and ds-DNA antibody levels in SLE patients, the results showed that TLR9 overexpression was positively correlated to IL-6 or ds-DNA antibody level during SLE development (for IL-6, *R* ^2^ = 0.768, for ds-DNA, *R* ^2^ = 0.730, Figures 2(c) and 2(d)).

### 3.3. Correlation between Silencing TLR9 and Cytokine Expression In Vitro

**Figure 3:**
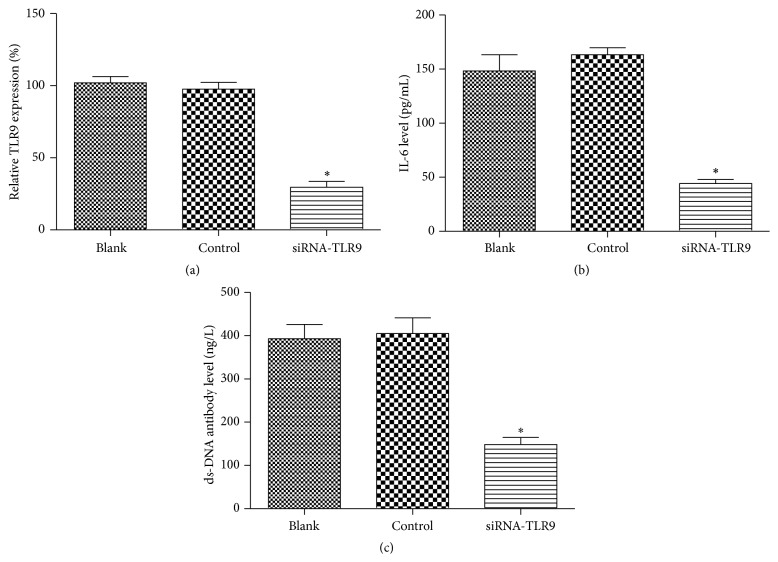
Correlation between silencing TLR9 and cytokine expressions in B cells from SLE patients. (a) TLR9 expression in each group of B cells; after being transfected with siRNA-TLR9, TLR9 in B cells was significantly declined; (b) IL-6 level in B cells transfected with siRNA-TLR9 plasma; (c) ds-DNA level in B cells transfected with siRNA-TLR9 plasma. ^*∗*^ *P* < 0.05, compared with the controls.

After being transfected with siRNA-TLR9 vector, TLR9 expression in SLE-isolated B cells was significantly declined compared with that in positive and negative controls (*P* < 0.05, Figure 3(a)). On the other hand, expressions of IL-6 and ds-DNA produced by B cells were also analyzed based on the RT-PCR; the results showed that IL-6 and ds-DNA levels in siRNA-TLR9 group were both significantly declined compared with the controls (*P* < 0.05, Figures 3(b) and 3(c)), indicating that silencing TLR9 may be positively correlated with IL-6 and ds-DNA levels in B cells.

### 3.4. Secretion of Cytokines Induced by TLR9 in B Cells

**Figure 4:**
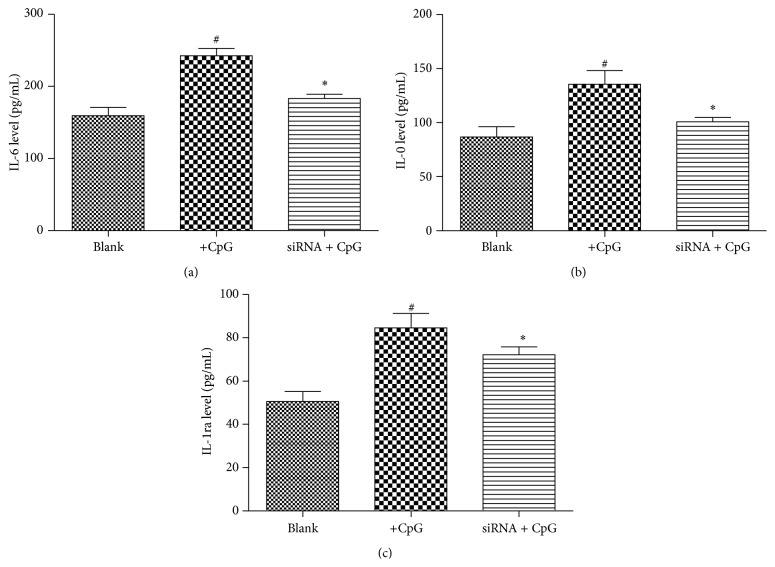
Effects of TLR9 expression on cytokines secretion in CpG stimulated B cells. (a)–(c) When SLE-isolated B cells were stimulated by CpG, secretions of IL-6, IL-10, and IL-1r*α* were significantly increased compared with the Blank group; however, this effect was reversed by siRNA-TLR9 transfection. Cells in Blank group were SLE-isolated B cells. ^#^ *P* < 0.05 compared with the Blank group, and ^*∗*^ *P* < 0.05 compared with the CpG group.

We further analyzed the cytokines including IL-6, IL-10, and IL-1r*α* levels for the identification of TLR9 expression on other cytokines secretion in SLE-isolated B cells (Figure 4). After being stimulated by CpG, secretion levels of IL-6, IL-10, and IL-1r*α* were all significantly increased compared to the control (Blank group; *P* < 0.05). Since TLR9 is the specific recognized receptor for DNA CpG in SLE cells, the results further showed that levels of the three factors were significantly decreased by the silenced TLR9 (*P* < 0.05), which suggests that silencing TLR9 was negatively correlated with the three-factor secretion in CpG stimulated B cells.

### 3.5. TLR9 Downstream Proteins Expression

**Figure 5:**
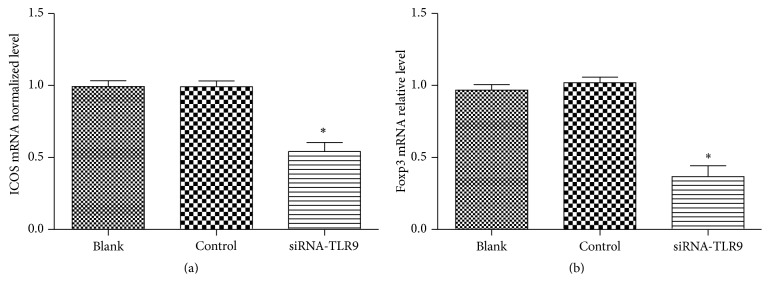
Influence of TLR9 expression on the downstream signals expression: (a) silenced TLR9 caused the downregulation of ICOS; (b) silenced TLR9 caused the downregulation of Foxp3. ^*∗*^ *P* < 0.05, compared with the controls.

The TLR9 downstream signal-related proteins expression including Foxp3 and ICOS was measured to further analyze the mechanism (Figure 5). Compared to the controls, relative mRNA levels of Foxp3 and ICOS (inducible costimulator) were both significantly decreased by the silenced TLR9 in vitro B cells, suggesting that silenced TLR9 may lead to the downregulation of pathway signals of Foxp3 and ICOS.

## 4. Discussion

Previous articles have reported the potential roles of TLR9 in SLE of animal body's development and progression; however, the results remain controversial. In this study, we used siRNA-mediated gene method to investigate the correlation of TLR9 expression cytokines secretion in SLE isolated in B cells. In agreement with former study [14], our data showed that TLR9 mRNA expression was higher in SLE patients than that in healthy persons (*P* < 0.05). Moreover, we firstly discovered that there was significant correlation between TLR9 expression and cytokines level including IL-6 and ds-DNA (*P* < 0.01). Furthermore, silencing TLR9 significantly decreased the secretion of IL-6, IL-10, and IL-1r*α* in SLE-isolated B cells, as well as the pathway-associated protein expression, including Foxp3 and ICOS.

Foregoing evidences have shown that TLR9 was involved in SLE through several manners such as mediating B-cell activation, mediating DC cells activation, and inducing cytokines secretion. For example, with the addition of IL-2 and IL-10 in CD27 labeled B cells, TLR9 expression in initial B cells was declined compared with that in memory B cells, which indicated the immune role of TLR9 in B-cell response [24]. Paper showed that that the structure change of dendritic cells (DC) may result in self-reactive B cells in SLE, and Means et al. described that TLR9 mRNA level was significantly increased when DC cells were stimulated by autoantibody-DNA complexes isolated from human SLE serum [25]. Also, TLR9 was correlated to induce cytokines production including TNF-*α* and IL-10 involved in SLE through activating pDC [13, 26]. All of these evidences showed that TLR9 was involved in maintaining the tolerance of B cell to SLE antibody and played a protective role for SLE patients.

In this study, secretions of IL-6 and ds-DNA antibody levels were declined by silencing TLR9, indicating that TLR9 expression was positively correlated to IL-6 and ds-DNA antibody secretion in B cells from SLE patients. IL-6 plays pivotal roles in inflammation and the maturation of B cells and has been proved to be closely related to SLE development and progression [27, 28], while ds-DNA antibody is a specific index for SLE diagnosis [29]. Influences of TLR9 expression on IL-6 and ds-DNA antibody levels in SLE have not been fully described. However, anti-ds-DNA antibody level was positively correlated with SLE disease activity [30], and radioimmunoassay had shown that anti-ds-DNA antibody in SLE patients at active stage was significantly high compared with that in SLE at resting stage [31]. On the other side, IL-6 mRNA level was high in SLE patients serum and can act as a biomarker in SLE development [32]. Based on our data, we speculated that TLR9 expression was positively correlated to SLE pathogen, and TLR9 expression could induce the B-cell secreted IL-6 and ds-DNA antibody to SLE serum. Therefore, TLR9 may be a biomarker in SLE diagnosis.

Subsequently, our study showed that ICOS and Foxp3 expressions were decreased by silencing TLR9 in B cells, suggesting that silencing TLR9 was negatively correlated ICOS and Foxp3 expressions in B cells. ICOS is a CD28 and CTLA-4 cell-surface receptor family protein, which functions in immunodeficiency biological processes. ICOS was overexpressed in peripheral blood from SLE patients and performed certain significant influence on Th2 type cytokine secretion, such as IL-10 and IL-4 [33]. It has been said that Foxp3 plays significant roles in SLE pathogen through being involved in the Treg cells at the transcriptional level [34]. Based on our data, we speculated that silencing TLR9 may be involved in SLE via downregulating the ICOS and Foxp3 signal pathway.

Meanwhile, Capolunghi and his colleagues have successfully blocked the self-antibodies produced in B cells from SLE patients using TLR9 inhibitor [35]. Contrary to Capolunghi's study, we used gene silenced method to investigate the associations between TLR9 expression and SLE development in human patients. Besides, Chen had demonstrated the understanding of TLR9 immune recognition in SLE development through gene knockdown method in SLE mice model [20]. However, our study has successfully illustrated the immune role of TLR9 in human SLE B cells using gene silenced method.

In conclusion, the data presented in this study suggests that TLR9 may play pivotal roles in SLE diagnosis through positively correlating with IL-6 and ds-DNA antibody secretion via being involved in the ICOS and Foxp3 signal pathway. The successful application of silencing TLR9 in human SLE B cells may loan theatrical basis for the possibility of TLR9 genetic therapy in SLE diagnosis and treatment. However, further studies are still needed to explore the deep mechanism.

## Figures and Tables

**Table 1:** Clinical characteristics for the patients enrolled in this study.

Information	Case group		Control group	
	Total	*P* value	Total	*P* value
Number of patients	39		27	
Age (years)^*∗*^	33.8 ± 14.7 (16–65)	*P* > 0.05	36.7 ± 15.1 (21–60)	*P* > 0.05
Gender (male/female)	9/30	*P* < 0.05	8/19	*P* < 0.05
Site				
Kidney	26			
Lung	9			
Heart	12			
Skin	15			
Stage				
Active stage (≥3)	27	*P* < 0.05		
Remittent stage (<3)	12			

^*∗*^Stands for the mean ± SEM (range).
